# Deciphering differences in microbial community characteristics and main factors between healthy and root rot-infected *Carya cathayensis* rhizosphere soils

**DOI:** 10.3389/fmicb.2024.1448675

**Published:** 2024-11-11

**Authors:** Wei Fang, Yiyang Zhu, Chenfei Liang, Shuai Shao, Junhui Chen, Hua Qing, Qiufang Xu

**Affiliations:** ^1^State Key Laboratory of Subtropical Silviculture, Zhejiang A&F University, Hangzhou, China; ^2^School of Environmental and Resource Sciences, Zhejiang A&F University, Hangzhou, China

**Keywords:** soil health, chemical properties, soil multifunctionality, pathogenic fungi, keystone species

## Abstract

**Introduction:**

Fusarium-induced root rot of *Carya cathayensis* (*C. cathayensis*) is a typical soil-borne disease that has severely damaged the *Carya cathayensis* industry in China. Understanding the interaction among soil microbial communities, soil characteristics, and pathogenic bacteria is very important for the ecological prevention and control of *Carya cathayensis* root rot.

**Methods:**

We used Miseq Illumina high-throughput sequencing technology to study the microbial community in the rhizosphere soil of healthy and diseased C. cathayensis, quantified the abundance of bacteria, fungi, and pathogenic fungi, and combined these with soil chemistry and enzyme activity indicators to analyze the characteristics of healthy and diseased rhizosphere soils.

**Results:**

We found that the pH, soil organic carbon(SOC), available nitrogen (AN), available phosphorus (AP), available potassium (AK),N-acetyl-*β*-D-glucosaminidase (NAG) β-glucosidase (BG), fungal gene copy number, bacterial community diversity and network complexity of the diseased soil were significantly lower (*p* < 0.05), while *Fusarium graminearum* copies number levels increased (*p* < 0.05). Additionally, the study found that healthy soils were enriched with beneficial bacteria such as *Subgroup_7* (0.08%), *MND1* (0.29%), *SWB02* (0.08%), and *Bradyrhizobium* (0.09%), as well as potential pathogen-suppressing fungi such as *Mortierella* (0.13%), *Preussia* (0.03%), and *Humicol* (0.37%), were found to be associated with the growth and development of *C. cathayensis*.

**Discussion:**

In summary, this research comprehensively reveals the differences in environmental and biological factors between healthy and diseased soils, as well as their correlations. It provides a theoretical basis for optimal soil environmental regulation and the construction of healthy microbial communities. This foundation facilitates the development of multifaceted strategies for the prevention and control of *C. cathayensis* root rot.

## Introduction

The *Carya cathayensis* (*Carya cathayensis* Sarg., 1916), belonging to the family of *Juglandaceae* and the genus *Carya Nutt.*, a valuable nut tree species endemic to China, is widely cultivated in provinces such as Zhejiang, Anhui, and Hunan, serving as a significant economic crop in these regions. However, due to its high economic value, the cultivation area of *C. cathayensis* has rapidly expanded in recent years. This expansion, coupled with the extensive use of chemical fertilizers and pesticides and excessive land management, has severely disrupted the forest ecosystem. Consequently, soil-borne root rot disease of *C. cathayensis* has frequently occurred on a large scale, with incidence rates reaching up to 90% in some areas, leading to widespread forest dieback. This situation poses a serious threat to the sustainable development and ecological safety of the *C. cathayensis* industry. Studies have shown that *C. cathayensis* root rot is mainly caused by *Fusarium oxysporum* (*F. oxysporum*) (Zhang et al., 2015) and *Fusarium graminearum* (*F. graminearum*) (Song, 2019). Current control measures are mainly based on chemical pesticides and agronomic practices, which are generally ineffective and, coupled with high costs of money and labor, make disease control very challenging. An imbalance in the soil microbial community is a major cause of root rot. Therefore, identifying the composition of microorganisms in diseased and healthy *C. cathayensis* soils, as well as the soil characteristics that affect these microorganisms, is crucial for managing root rot in these soils.

The composition and function of soil microorganisms are regulated by soil properties, including soil pH, organic carbon (SOC) content, nutrient concentrations, and the activity of soil extracellular enzymes (EEAs) (Zhou et al., 2020). Research indicates that these properties are sensitive to diseases and have negative impacts on microbial abundance, diversity, and composition. For example, long-term intensive management has led to soil acidification and nutrient imbalance in *C. cathayensis* soils, with stronger acidification correlating with more severe root rot disease (Zhang et al., 2011). Similarly, the accumulation of organic acids from plant roots can lower soil pH, while the accumulation of root exudates (phenolic acids and alcohols) enriches pathogens, increasing the abundance of rhizosphere pathogens such as *F. oxysporum* and *Talaromyces helice*s (Liu et al., 2020; Wu et al., 2020). In contrast, the nutrient and organic carbon content in the rhizosphere soil of healthy plants is significantly higher than in diseased soil (*p* < 0.05). Qi et al. (2019) found that the chemical properties and microbial networks of healthy soils and bacterial wilt-infected soils showed that diseased soils had lower available nitrogen, phosphorus, potassium, and organic carbon compared to healthy soils, and the microbial network in healthy soils was more complex and stable. Furthermore, studies indicate that soil extracellular enzyme activity is closely related to soil microbial activity and negatively correlated with disease severity (Chun et al., 2014), often serving as a critical indicator for predicting soil-borne diseases (Chuan et al., 2011), providing a comprehensive reflection of the functional characteristics of soil microbial communities. Therefore, a systematic understanding of the impact of diseases on various soil properties (i.e., soil multifunctionality, SMF) is necessary. Additionally, there is a positive linear relationship between soil ecosystem functions and the diversity and composition of soil microbial communities (Mori et al., 2018). Further consideration of the relationship between the soil microbiome, particularly characteristic microorganisms, and soil functions is required.

Plants live within microbial communities, forming a complex assemblage of various microorganisms in the rhizosphere environment (Qu et al., 2020). However, under high-intensity intensive management, the enrichment or reduction of certain rhizosphere microorganisms can negatively impact the diversity or composition of the rhizosphere micro-ecosystem, leading to the occurrence of soil-borne diseases. For example, the increased abundance of pathogenic fungi such as Fusarium triggers diseases in hundreds of plants (Dong and Fang, 2021) including *bananas*, *wheat*, and *watermelon*, while the reduced abundance of *Firmicutes* and *Actinobacteria* in the *tomato* rhizosphere can lead to the outbreak of bacterial wilt (Lee et al., 2021). Soils with highly abundant beneficial microorganisms can more effectively suppress pathogens and maintain plant health (Xiong et al., 2017), such as *Pseudomonas* and *Bacillus*, and *Trichoderma*, which have been proven to inhibit soil-borne pathogens (Haas and Défago, 2005; Mazurier et al., 2009; Ozimek and Hanaka, 2021). However, studies indicate that the pathogen suppression effect of these beneficial bacteria often fails to fully manifest as in laboratory conditions (Maraha et al., 2004; Mazzola and Freilich, 2017). Researchers attribute this to the difficulty of the introduced biocontrol bacteria in competing for resources with indigenous soil microorganisms or the unsuitability of the soil environment for the survival of the introduced microorganisms (Maraha et al., 2004). Therefore, conducting *in-situ* studies of microbial communities in healthy and diseased soils, and artificially constructing synthetic communities for reintroduction into soil, has become the most effective approach for the microbial biocontrol of soil-borne diseases. Therefore, it is crucial to understand the characteristics of the microbial communities in the rhizosphere soil of healthy and diseased *C. cathayensis,* as well as how to activate beneficial microorganisms, which is key to addressing the issue of root rot.

Early research on root rot in *C. cathayensis* primarily focused on pathogen control experiments and the structure of the bacterial microbial community in the soil, neglecting the characteristic microorganisms of the entire microbial community (both bacteria and fungi), their association with soil properties, and their impact on the occurrence of root rot. Furthermore, the potential of soil microorganisms in promoting crop growth and disease prevention has not been fully explored. Therefore, comprehensive studies are needed to assess the chemical properties and extracellular enzyme activities of *C. cathayensis* rhizosphere soil, and to deeply analyze the dynamic changes in microbial communities and their relationships with soil chemical properties between healthy and diseased soils.

Based on the limitations of existing research, by employing Miseq Illumina high-throughput sequencing technology to comprehensively analyze the microecological characteristics of healthy and diseased soils from multiple dimensions, including microbial community diversity, complex network structure, and soil physicochemical properties. And identify key microbial taxa that indicate soil health, and investigate their potential as microbial biocontrol agents. These findings not only enhance our understanding of the changes in soil microbial communities during the occurrence of *C. cathayensis* root rot, but also will provide a theoretical basis for preventing and controlling *C. cathayensis* root rot, as well as for developing new microbial biocontrol agents strategies.

## Materials and methods

### Study area overview

The study area is located in Tuankou Town, Lin’an District, Hangzhou City, Zhejiang Province (119°16′N, 30°03′E), which is a core production area for *C. cathayensis* in China. This region falls within the subtropical monsoon climate zone, with an average annual temperature of 17.2°C. The soil in the study area is predominantly lateritic, with an elevation ranging from 50 m to 1,100 m. In September 2019, we conducted a field survey of the area to assess the incidence of root rot disease in *C. cathayensis* forests and selected nine areas with healthy *C. cathayensis* trees and nine areas with trees affected by root rot disease for sample collection (The roots of many *C. cathayensis* trees in this area have turned black, rotted, and even died). Each area measured 20 m × 20 m. Soil samples were collected using a standardized five-point sampling method, employing root-shaking to gather soil from the rhizosphere of *C. cathayensis* at a depth of 5–20 cm, evenly distributed across five locations within each plot. From each sampling point, a composite soil sample (approximately 500 g) was collected by pooling soil from around the roots of *C. cathayensis*. The collected soil from each plot was thoroughly mixed to create one composite sample per plot, resulting in a total of 18 composite samples (9 healthy and 9 diseased plots). The collected soil samples were thoroughly mixed, sieved (2 mm mesh size), and placed into sterile sealed bags, totaling 18 samples. These soil samples were divided into three parts for different analyses: one part of the fresh soil samples was used to determine extracellular enzyme activity; another part was freeze-dried and stored in a −80°C freezer for subsequent extraction of soil microbial DNA; the remaining soil samples were air-dried for analysis of other soil properties.

### Soil chemical properties and extracellular enzyme activity assays

The analysis of basic chemical properties of air-dried soil was performed according to “Methods of Soil Agricultural Chemical Analysis” (Lu, 1999), assessing five key indicators: soil organic carbon (SOC), alkali-hydrolyzable nitrogen (AN), available phosphorus (AP), available potassium (AK), and pH value. For fresh soil, the activities of seven enzymes involved in the cycles of carbon (C), nitrogen (N), and phosphorus (P) were tested: *α-glucosidase* (AG), *β-glucosidase* (BG), *cellobiohydrolase* (CB), *leucine aminopeptidase* (LAP), *N-acetyl-β-glucosaminidase* (NAG), *acid phosphatase* (PHOS), and *β-xylosidase* (XYL). These seven soil hydrolase activities, indicative of soil quality and health, were assessed using a fluorescence microplate assay technique (Saiya-Cork et al., 2002). Specifically, 2 g of fresh soil was placed in a 50 mL centrifuge tube, followed by the addition of 30 mL of ammonium acetate buffer (pH 5.0) and shaking at 180 rpm at 25°C for 30 min. The remaining soil solution in the centrifuge tube was then transferred to a beaker with 70 mL of ammonium acetate buffer and stirred using a magnetic stirrer at 1.25 rpm for 1 min. Using a pipette, 200 μL of the soil suspension was transferred to a 96-well plate, followed immediately by the addition of 50 μL of the reaction substrate. The plate was incubated at 25°C in the dark for 3 h, followed by the quick addition of 15 μL of 0.5 mol·L^−1^ sodium hydroxide to terminate the reaction. Absorbance values were measured using a multifunctional enzyme label meter to calculate soil enzyme activity.

### DNA extraction, amplicon sequencing, and qPCR

Total genomic DNA was extracted from 0.25 g soil samples using the Power Soil DNA Isolation Kit. DNA quality and quantity were assessed via spectrophotometry (QuickDrop2017) and agarose gel electrophoresis. The extracted DNA was aliquoted and stored at −80°C for future use. Soil genomic DNA analysis was conducted through real-time quantitative PCR (qPCR) and high-throughput sequencing. For bacteria, the V3-V4 regions were amplified using the F338/806R primers (Ding et al., 2020), and for fungi, the ITS1 region was amplified using the ITS1F/ITS2 primers (May et al., 2001) for paired-end sequencing on the Illumina MiSeq platform (Illumina Inc., San Diego, CA, United States).

Quantitative PCR was used to measure the abundance of soil bacteria, fungi, and the pathogenic fungi *F. oxysporum* and *F. graminearum*, with each sample tested in triplicate. The fungal-to-bacterial ratio (F: B) was calculated using the total gene abundance of soil bacteria and fungi obtained. *F. oxysporum* was targeted with the Fo1/Fo2 primers (Wagan et al., 2002), and *F. graminearum* with the Fg16F/Fg16R primers (Krnjaja et al., 2018). The qPCR reaction mix was 20 μL, consisting of 1 μL DNA template, 10 μL premix SYBR, 0.5 μL of each forward and reverse primer, and 8 μL ddH2O. The conditions for the quantitative PCR are listed in Supplementary Table S1.

### The bioinformatics analysis of high-throughput sequencing data

High-throughput FASTQ format raw sequencing data were processed using USEARCH v.10.0 (Edgar, 2010), VSEARCH v.2.13 (Rognes et al., 2016), and internal scripts (Zhang et al., 2018). Through these tools, strict quality control was implemented to ensure an error rate below 1%. Subsequently, data denoising was performed using unoise3, along with assembly and chimera removal operations, to enhance the accuracy and reliability of the data. Finally, clustering at 97% similarity was conducted to obtain the final Amplicon Sequence Variant (ASV) abundances and representative sequences for subsequent analyses. For species annotation, the-sintax command of VSEARCH was used in conjunction with the RDP database (Cole et al., 2014) for bacteria and the UNITE database for fungal species annotations (Abarenkov et al., 2010).

### Statistical analysis

One-way ANOVA was used to determine the differences in soil chemical properties and enzyme activities between the rhizosphere soils of healthy and diseased plants. Alpha diversity indices were calculated using the vegan package, and the EasyStat package (version: 0.1.0^1^) was employed for testing and plotting. We performed Principal Coordinates Analysis (PCoA) using the Bray-Curtis distance matrix to visualize the differences in microbial community structure between healthy and diseased *C. cathayensis* soils. Analysis of Similarities (ANOSIM) was conducted to test for significant differences, and plots were generated using ggplot2 (Abdi and Williams, 2010). To explore the changes in community assembly in diseased soil, a null model was used to quantify the deviation of the community’s observed phylogenetic distance from the expected random phylogenetic distance, determining whether community changes were dominated by deterministic or stochastic processes. The βNTI was calculated using the R packages picante and NST, as referenced in the “Microbiome Experimental Handbook”.^2^

Network analysis was performed using the ggClusterNet package (version 0.1.7^3^) for computation and visualization. The default threshold was set to display relationships with a correlation coefficient (R) greater than 0.8 and a *p*-value less than 0.05. Microbes with a relative abundance greater than 0.5% were selected for network relationship exploration, and the network’s topological characteristics were determined. The network diagram was color-filled according to the module, and the ggClusterNet package was also used to calculate the proportion of negative correlations and the network’s vulnerability, with the default threshold set to a correlation coefficient *R* > 0.8 and *p* < 0.05.

Machine learning was conducted using the R package randomForest, along with other packages such as tsne, to identify characteristic microbes that are significantly different and important within the microbial community, with visualization performed using ggplot2 (Banerjee et al., 2019). Finally, a Mantel Test matrix analysis was carried out to analyze the correlations between microbial biomarkers in healthy and diseased soils identified by random forest (RF) and soil chemical properties, enzyme activities, as well as microbial copy numbers quantified by qPCR. *p* < 0.05 was used to determine the key factors influencing the microbial community.

Three different methods—the mean, single threshold, and multiple threshold methods—were used to quantify soil multifunctionalit (SMF) (Byrnes et al., 2013). All soil measurement indicators (including soil pH, SOC, AN, AP, AK, and soil enzyme activities AG, BG, CB, LAP, NAG, PHOS, XYL) were standardized using Z-score transformation (Jucker and Coomes, 2012). Z-scores for individual functions were averaged to calculate a soil multifunctionality index for each sample. Microbial diversity was assessed using the Simpson index for bacterial and fungal communities, applying a method similar to that used for calculating the Z-scores. The single threshold method evaluated if multiple functions were performed at a high level simultaneously and exceeded a specified threshold percentage of the maximum function (Mori et al., 2016). We set single thresholds at 20, 40, 60, and 80% and used a generalized linear model to determine if the impact of microbial diversity on soil multifunctionality varies across these thresholds. The correlation between microbial diversity and the total number of functions was analyzed using the multiple threshold method with functionalities from the “multifunc” package (Byrnes et al., 2013). Finally, the relationships between soil multifunctionality and microbial diversity, network complexity, and microbial absolute and relative abundances were tested using linear regression. In addition, soil enzyme activities belonging to the same functional group were normalized using an equation approach (Luo et al., 2018).

## Results

### Chemical properties and enzyme activity of soil

**Figure 1:**
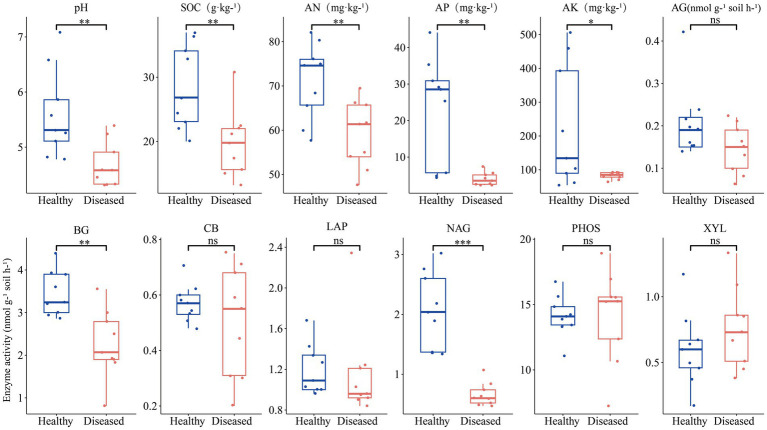
Chemical properties and enzyme activity of rhizosphere soil from healthy and diseased *Carya cathayensis* trees. ***, **, and * indicate statistically significant differences between healthy and diseased *Carya cathayensis* rhizosphere soil at *p* < 0.001, *p* < 0.01, and *p* < 0.05, respectively, while “ns” denotes no statistical difference between the two groups.

In terms of soil chemical properties (Figure 1), the soil from diseased sites showed significantly lower levels of pH, SOC, AN, AP, and AK compared to the soil from healthy plant sites. Regarding enzyme activity, the rhizosphere soil of healthy *C. cathayensis* trees exhibited significantly higher activities of AG, BG and NAG enzymes compared to the rhizosphere soil of diseased *C. cathayensis* trees.

### Microbial gene abundance and α and β diversity in the soil microbiome

**Figure 2:**
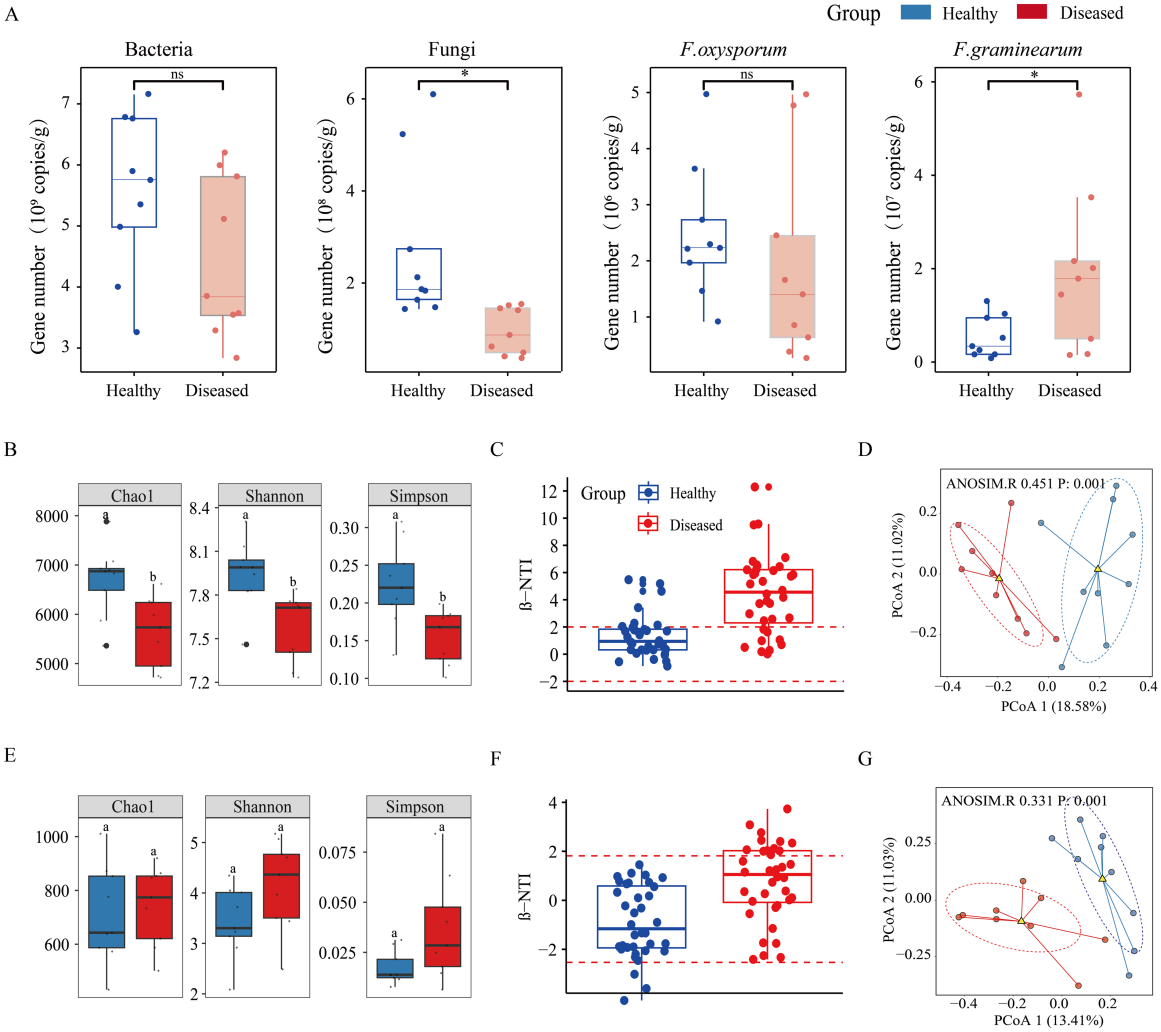
Microbial abundance and *α* and β diversity in the rhizosphere soil of healthy and diseased *Carya cathayensis.* **(A)** qPCR quantification of the abundance of soil bacteria, fungi, *F. oxysporum*, and *F. graminearum*. **(B,E)** Represent the Chao1 index, Shannon index, and Simpson index for bacterial and fungal communities, respectively. **(C,F)** Represent the βNTI phylogenetic diversity index for bacterial and fungal communities, respectively. **(D,G)** Represent Principal Coordinates Analysis (PCoA) for bacterial and fungal communities, respectively. * indicates a significant difference between healthy and diseased *Carya cathayensis* rhizosphere soil, while ns indicates no difference at the 0.05 level. p: 0.001 indicates a significant difference between the rhizosphere soil of healthy and diseased *Carya cathayensis*.

Quantitative analysis of microbial abundance revealed that both bacterial and fungal gene abundances in the rhizosphere soil of diseased *C. cathayensis* trees were significantly reduced, with fungal gene copy numbers notably lower than in healthy soil. Pathogen quantification showed that the abundance of *F. graminearum* genes was significantly higher in the rhizosphere soil of diseased *C. cathayensis* compared to healthy soil (*p* < 0.05; Figure 2A). The Chao1 index, Shannon index, and Simpson index were employed to estimate microbial richness and diversity. Compared to healthy soil, diseased soil exhibited significant differences in bacterial richness and diversity (*p* < 0.05; Figure 2B), whereas no difference was observed in fungal richness and diversity between diseased and healthy soils (Figure 2E). To better quantify the phylogenetic distance between microbial communities in diseased and healthy soils, we calculated the turnover of microbial community phylogeny in both healthy and diseased soils (Figures 2C,F). The results indicated that the bacterial *β*-NTI values in healthy soil were mainly concentrated within the-2 to 2 interval, suggesting that changes in bacterial community structure were mainly influenced by stochastic factors. In contrast, bacterial β-NTI values in diseased soil were primarily distributed in intervals greater than 2, indicating that changes in the bacterial community structure of diseased soil were mainly driven by deterministic factors. The β-NTI values for fungal communities in both healthy and diseased *C. cathayensis* rhizosphere soils were primarily within the-2 to 2 interval, suggesting that changes in fungal community structure were mainly influenced by stochastic factors. Principal coordinates analysis (PCoA) based on Bray-Curtis distance (Figures 2D,G) revealed significant differences between bacterial and fungal communities in healthy and diseased *C. cathayensis* rhizosphere soils, with separation along the first principal axis, indicating that the greatest differences between the two groups were likely due to disease.

### Soil microbial co-occurrence network

**Figure 3:**
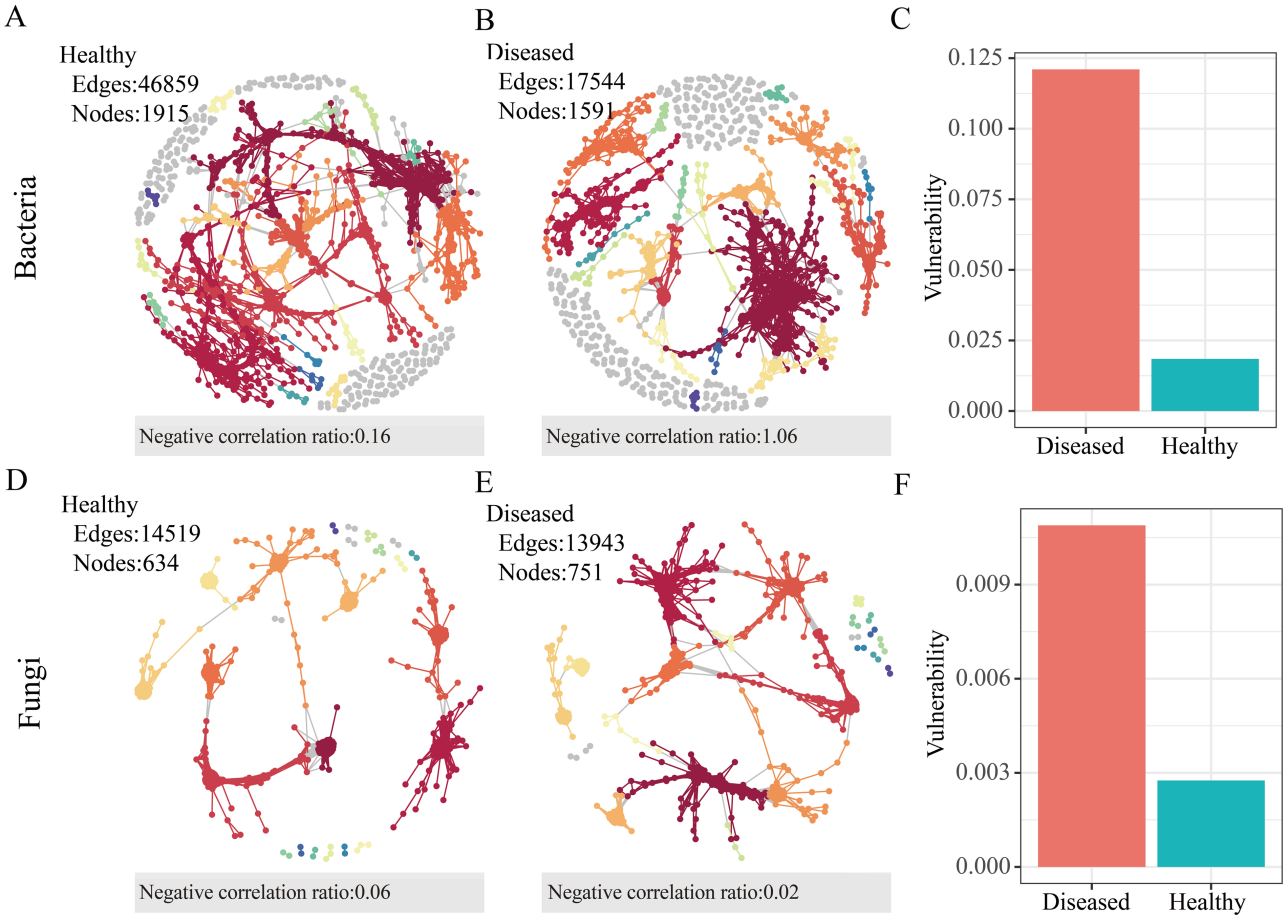
Molecular ecological networks of microbial associations in the rhizosphere soil of healthy and diseased *Carya cathayensis.* **(A)** Bacterial network in the rhizosphere soil of healthy *Carya cathayensis.* **(B)** Bacterial network in the rhizosphere soil of diseased *Carya cathayensis.* **(C)** Vulnerability of bacterial networks in the rhizosphere soil of healthy and diseased *Carya cathayensis.* **(D)** Fungal network in the rhizosphere soil of healthy *Carya cathayensis*. **(E)** Fungal network in the rhizosphere soil of diseased *Carya cathayensis*. **(F)** Vulnerability of fungal networks in the rhizosphere soil of healthy and diseased *Carya cathayensis.*

To quantify the differences between the microbial networks in the rhizosphere soil of healthy and diseased *C. cathayensis*, the topological characteristics of the co-occurrence networks were calculated. In the bacterial network, healthy soil possesses 1,915 nodes and 46,859 edges, more than those in diseased soil (Figures 3A,B), but the diseased soil network exhibited a higher rate of negative correlations. In the fungal networks (Figures 3D,E), the number of nodes and edges in the microbial communities of healthy and diseased *C. cathayensis* rhizosphere soil was similar, with the fungal network in healthy *C. cathayensis* rhizosphere soil showing a higher rate of negative correlations between nodes. The network vulnerability results (Figures 3C,F) indicate that the vulnerability of bacterial and fungal networks in healthy *C. cathayensis* rhizosphere soil is significantly lower than that in diseased soil. Lower vulnerability corresponds to higher network stability, suggesting that the stability of bacterial and fungal microbial networks in healthy soil is greater than in diseased soil.

### Microbial characteristics of healthy and diseased soil and analysis with environmental factors

**Figure 4:**
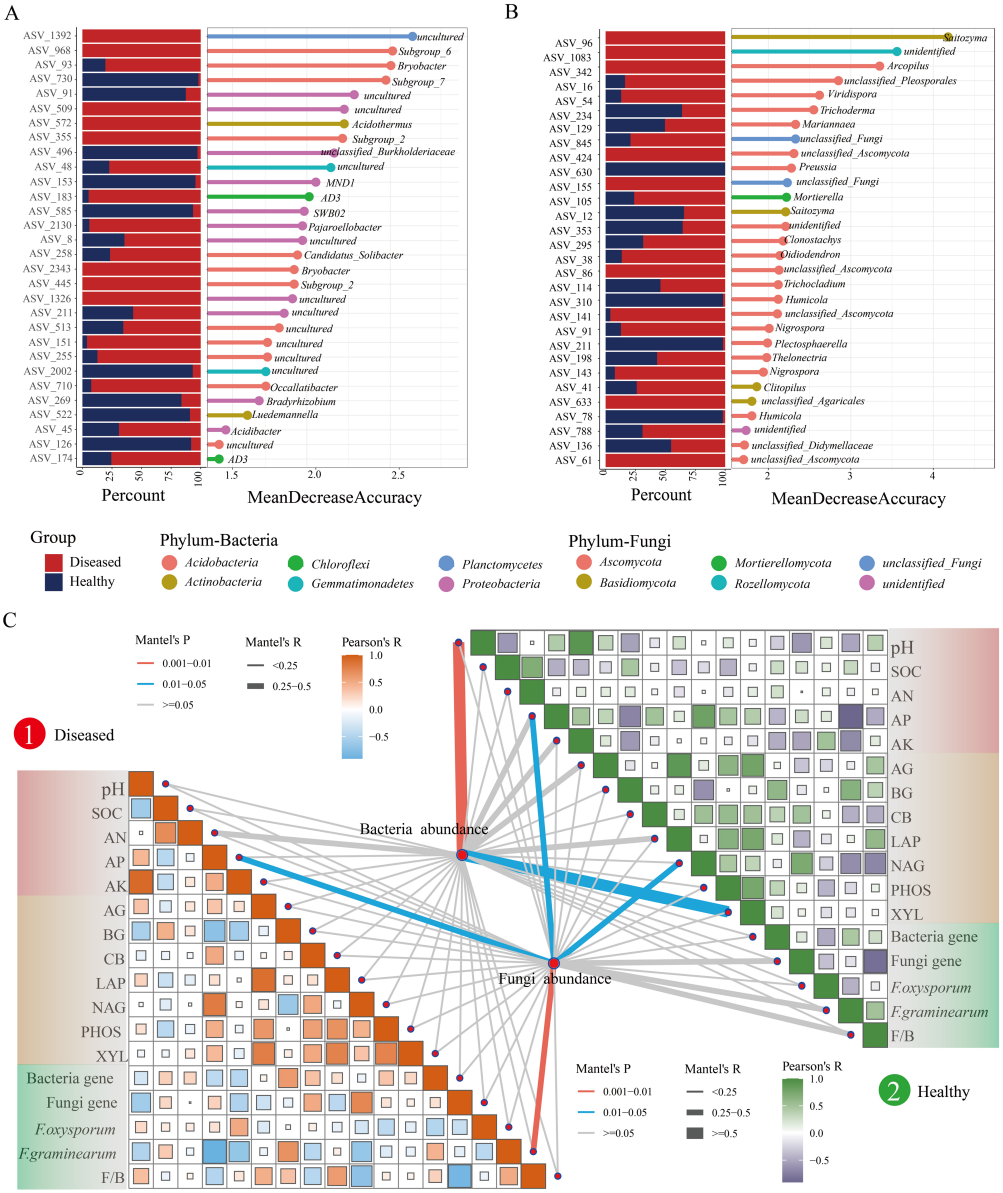
Machine learning extraction of microbial community characteristics in the rhizosphere soil of healthy and diseased *Carya cathayensis* and their correlation with environmental factors. **(A,B)** The relative proportion of the feature asv (a for bacterial, b for fungal) in healthy and diseased samples are displayed. **(C)** Characteristic ASV and environmental feature Mantel Test matrix analyses.

To distinguish the microbial communities in the rhizosphere soil of healthy and diseased *C. cathayensis*, a Random Forest (RF) classifier was built based on genus-level data for learning and classification of healthy and diseased soils. The results indicated an accuracy of 88.89% for bacteria and 83.33% for fungi. A total of 204 bacterial ASVs and 126 fungal ASVs were identified, which were used as biomarkers (Supplementary Tables S2-1, S2-2). Within the bacterial communities, these biomarkers mainly belonged to *Acidobacteria* and *Proteobacteria*. For the fungal communities, biomarkers predominantly belonged to Ascomycota and Basidiomycota. Notably, Fusarium ASVs 29 and 302 were more abundant in diseased soils. To visually present the differences in microbial community characteristics between healthy and diseased *C. cathayensis* rhizosphere soils, the top 30 important biomarkers were prioritized for display (Figures 4A,B). The results showed that *Subgroup_7* (0.08%), *MND1* (0.29%), *Bradyrhizobium* (0.10%), and *Luedemannella* (0.08%) were dominant in the bacterial community of healthy soil, while *Preussia* (0.03%) and *Humicola* (0.05%) genera were prevalent in the fungal community of healthy soil. In the bacterial community of diseased soil, *Subgroup_6* (0.06%), *Bryobacter* (0.30%), *Acidothermus* (0.08%), *Subgroup_2* (0.11%)*, AD3* (0.21%), and *Occallatibacter* (0.08%) were dominant, whereas in the fungal community of diseased soil, *Saitozyma* (0.55%)*, Arcopilus* (0.09%)*, Viridispora* (0.96%)*, Mortierella* (0.13%)*, Oidiodendron* (1.47%)*, Nigrospora* (0.60%), and *Clitopilus* (1.36%) were identified as dominant biomarkers.

Subsequently, we conducted Mantel Test matrix analyses on these microbial biomarkers in relation to environmental factors (Figure 4C). We discovered that in the microbial communities of diseased soil, the abundance of biomarkers showed significant correlations with AP and *F. graminearum*, with the correlation with *F. graminearum* reaching a highly significant level (*p* < 0.01). In healthy soil, bacterial biomarkers were related to pH and XYL with the correlation with pH also being highly significant, while the abundance of fungal microbes was related to AP and NAG (*p* < 0.05). Furthermore, considering whether soil properties affect microbial biomarkers or the entire microbial community, we analyzed the correlation between environmental factors and the entire microbial community in the rhizosphere soil of healthy and diseased *C. cathayensis* (Supplementary Figure S1). The results were consistent with the microbial biomarkers obtained from the random Forest analysis, indicating that the differences in the microbial communities between healthy and diseased *C. cathayensis* rhizosphere soils are primarily influenced by these factors.

### Relationship between soil multifunctionality and microorganisms

**Figure 5:**
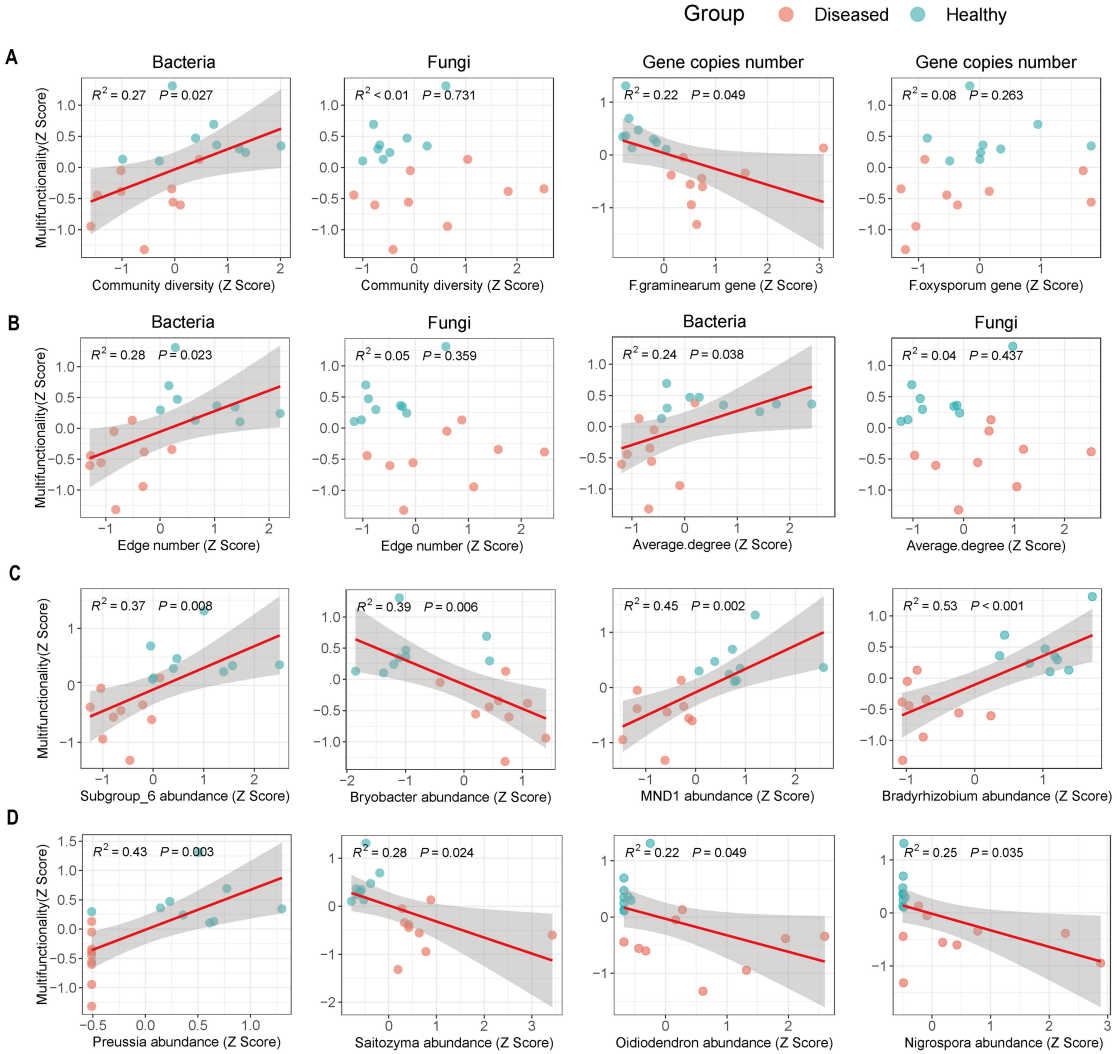
Relationship between soil multifunctionality and microorganisms. **(A)** Represents the relationships of bacterial community diversity, fungal community diversity, *Fusarium graminearum*, and *Fusarium oxysporum* with soil multifunctionality. **(B)** Represents the relationship between soil multifunctionality and the complexity of bacterial and fungal networks (number of edges and average degree). **(C)** Represents the relationship between soil multifunctionality and bacteria markers. **(D)** Represents the relationship between soil multifunctionality and fungal markers. The red solid line indicates relationships tested by linear regression, and the shaded area represents the 95% confidence interval of the fit.

The relationship between soil microorganisms and multifunctionality shows that the multifunctionality of diseased soils is significantly reduced (Supplementary Figure S2). Linear regression analysis (Figure 5) indicates that soil multifunctionality is significantly positively correlated (*p* < 0.05) with bacterial community diversity, bacterial network complexity (Figures 5A,B) (nodes and average degree), and beneficial microorganisms (Figures 5C,D) (*Subgroup_6, MND1, Bradyrhizobium, Preussia*). Conversely, there is a significant negative correlation (*p* < 0.05) with the abundance of *Fusarium graminearum* (Figure 5A) and the abundance of beneficial microorganisms (*Bryobacter, Saitozyma, Oidiodendron, Nigrospora*) (Figures 5C,D). In contrast, the diversity and network complexity of the fungal community do not show a clear correlation with soil multifunctionality (Figures 5A,B; Supplementary Figures S3A–D).

## Discussion

Long-term excessive management has led to the accumulation of soil-borne pathogens and nutrient depletion, disrupting plant–soil feedback loops, and promoting disease outbreaks, ultimately threatening the sustainability of *C. cathayensis* forests. Therefore, understanding the composition of rhizosphere microbial communities and their relationship with environmental factors is crucial for the effective control of soil-borne diseases. This study identifies specific microbial indicators by analyzing key microbial communities and their subgroups that drive these changes. These indicators have the potential to serve as early warning tools for disease detection and soil health monitoring, helping to detect and prevent soil health issues in a timely manner.

Intensive management practices have significantly altered soil properties and microbial communities, leading to the accumulation of soil-borne pathogens and depletion of essential nutrients. These changes create conditions conducive to disease outbreaks, thereby threatening the health and sustainability of *C. cathayensis* forests. Research indicates significant reductions in soil pH, SOC, AN, AP, and AK levels in diseased soils, aligning with prior findings (Liu et al., 2021). Intensive management practices, including the excessive application of nitrogen fertilizers and the accumulation of phenolic compounds, can reduce soil pH, thereby exacerbating the occurrence of soil-borne diseases. Organic matter serves as the most basic source of energy and carbon for soil organisms, and soils rich in organic matter can support a more diverse population of beneficial and antagonistic microbes. It is generally believed that soil organic matter primarily originates from plant litter and residues. Diverse plant species contribute varied organic materials to the soil, catering to the preferences of different microbes and thus preserving microbial diversity (Štursová and Baldrian, 2011), thus maintaining soil microbial diversity. However, under long-term intensive management, the continuous removal of other vegetation in *C. cathayensis* forests results in fewer plant residues and a shortage of available organic matter in the soil. Additionally, because *C. cathayensis* forests are located in mountainous and hilly areas with relatively loose soil texture, excessive management can also exacerbate soil erosion, leading to nutrient loss. Studies show that AP and AK levels drop significantly in diseased soils; phosphorus-rich soil usually enhances root growth, helping plants resist pathogens and diseases (Dordas, 2008). Similarly, the abundance of available potassium in the soil can inhibit many soil-borne diseases in crops. The mechanism is that sufficient available potassium in the soil ensures the supply of potassium nutrition in plant tissues, thereby enhancing plant resistance to pathogens (Huber et al., 2012).

Soil enzymes are vital for the growth and metabolism of microbial communities. Our data show that NAG enzyme activity is higher in healthy soil than in diseased soil. Previous research has demonstrated that NAG in the rhizosphere can inhibit fungal diseases, with its involvement in the conversion of ammonium to ammonia being one of the most common mechanisms for killing pathogens (Durán et al., 2018). Furthermore, NAG suppresses fungal diseases by degrading fungal cell walls, serving as a key protein in pathogen-host interactions (Graham and Strauss, 2021). Consequently, we hypothesize that soil in the rhizosphere of healthy plants contains more microorganisms capable of producing NAG, which degrades pathogenic fungi cell walls and combats infections. BG enzyme activity indicates the rate of organic matter decomposition and microbial activity in the soil. It breaks down BG compounds in plant residues, root exudates, and other organic wastes into glucose, crucial for microbial use and vital for soil nutrient cycling, carbon supply, and stress defense (Dong and Fang, 2021). Similarly, the BG enzyme activity is lower in the rhizosphere soil of diseased *C. cathayensis* trees. In contrast, the rhizosphere soil of healthy *C. cathayensis* trees, richer in organic matter and microbial activity, enables microbes to compete with pathogens, inhibiting root rot development.

Rhizosphere microbial communities play a crucial role in promoting crop growth and maintaining crop health (Dehnen-Schmutz et al., 2010). However, certain rhizosphere microbes can also negatively impact the diversity or composition of the rhizosphere soil microbiota, leading to the occurrence of soil-borne diseases (Gu et al., 2022). In soils subjected to excessive management, soil properties (including chemical properties and enzyme activity) are important factors affecting the incidence of diseases, disrupting the abundance of microbes (Yuan et al., 2020). This study has shown that the gene abundance of bacteria and fungi in the rhizosphere of healthy plants is higher than in diseased soil, especially the fungal gene abundance in diseased soil is significantly lower than in healthy soil, while the gene abundance of *F. graminearum* is significantly higher in diseased soil. This indicates that disease infection leads to a decrease in the total microbial gene abundance in the soil, particularly affecting the fungal gene abundance more significantly. The analysis of microbial community diversity indices revealed that the bacterial community diversity in the rhizosphere soil of healthy *C. cathayensis* is higher, suggesting that disease leads to a reduction in microbial community *α*-diversity, causing an imbalance in the bacterial microbial community equilibrium, but not significantly affecting the total gene abundance of bacteria, more so affecting the diversity of the bacterial microbial community. Similarly, disease also affects the phylogeny of microbial communities (Gao et al., 2021). We found that the assembly of the bacterial community in the diseased rhizosphere soil is influenced by deterministic processes, meaning that deterministic processes play a dominant role in the assembly of bacterial communities in diseased soil, exerting a selective effect on community assembly. In contrast, stochastic processes play a greater role than deterministic processes in the construction of fungal communities. This may be due to the influence of rhizosphere soil properties on the assembly of rhizosphere microbial communities, with existing studies showing that disease can change the assembly process of rhizosphere microbial communities (Morriën et al., 2017), which is consistent with our conclusions.

Microbes do not grow in isolation but form complex networks of associations (Morriën et al., 2017). Microbial co-occurrence networks can reflect the direct and indirect connections between coexisting microbes in the environment, providing insights into the functions of microbial communities and the ecological niches occupied by microbes (de Vries et al., 2018). Consistent with the results of Wu C. et al. (2021) and Wu Y. et al. (2021), compared to the rhizosphere soil of healthy *C. cathayensis*, the bacterial network in the rhizosphere soil of diseased plants shows lower complexity, with fewer nodes and edges, poorer network stability, and a higher proportion of negative correlations. This indicates that the bacterial microbial community in diseased soil is more vulnerable. Conversely, in the rhizosphere soil of healthy *C. cathayensis*, the connections between microbes are stronger and closer, with higher connectivity and stability, making it more likely to effectively inhibit the invasion of pathogens (Tan et al., 2021). Therefore, based on the microbial diversity indicators in this study, the response of bacterial communities to disease is greater than that of fungal communities, making bacterial community parameters possibly more suitable as indicators for assessing the health and disease of *C. cathayensis* rhizosphere soil.

Furthermore, we also utilized the Random Forest (RF) model at the genus level to distinguish between microbial community characteristics of diseased and healthy soils. The results indicated that microbial features with dominant abundance were more frequently classified into diseased soil, including some genera that were not annotated. In the bacterial community of diseased soil, Subgroup_6, *Bryobacter, Acidothermus*, *Subgroup_2, AD3, Subgroup_5, Occallatibacter*, and others had a dominant presence, most of which belong to *Acidobacteria*. The pH value has a significant impact on the diversity of *Acidobacteria*, with numerous studies showing that within a certain range, the overall abundance of *Acidobacteria* is inversely proportional to pH value (Fierer et al., 2007; Lauber et al., 2008). This is because microbes in the *Acidobacteria* phylum grow slowly and can easily be replaced by faster-growing microbes when soil nutrients or structure changes (Ward et al., 2009; Liu et al., 2016), and in diseased soil, the abundance of other bacteria decreases due to pH effects, thus increasing the abundance of *Acidobacteria* microbes.

In the fungal microbial community of diseased soil, saprophytic fungi such as *Saitozyma, Arcopilus, Viridispora, Oidiodendron, Nigrospora, Clitopilus*, etc., were enriched in the rhizosphere soil and play a significant role in the decomposition of organic carbon and may influence the structure of other microbial communities in the soil environment. For instance, *Oidiodendron* has been reported as a mycorrhizal fungus, which can form symbiotic relationships with plant roots, aiding in nutrient uptake and stress resistance (Wu C. et al., 2021; Wu Y. et al., 2021). Similarly, *Saitozyma* has been reported as a beneficial fungus that promotes plant growth and enhances disease resistance (Zhou et al., 2021), while Nigrospora has been suggested to possess pathogenic potential under certain environmental conditions (Liu et al., 2024). The enrichment of these fungal groups in diseased soils may be driven by changes in soil conditions (e.g., pH, nutrient content, and microbial imbalance) and may have an indirect impact on the development of root rot or influence disease progression by altering the rhizosphere microbial dynamics. Additionally, it has been reported that systems with higher soil saprophytic fungal diversity have higher plant productivity stability (Liu et al., 2022). Contrary to our findings, the organic carbon content in diseased soil significantly decreased, suggesting that the extensive activity of this group of fungi is related to the massive death of *C. cathayensis* roots, which are enriched in the rhizosphere. This also corresponds with our sampling observations, where diseased *C. cathayensis* roots exhibited severe rot. Notably, the genus *Mortierella* was also enriched in the rhizosphere soil of diseased *C. cathayensis.* This genus is known for promoting plant growth and protecting plants from diseases and abiotic stress through various mechanisms (Ozimek and Hanaka, 2021). Research by Feng et al. also found that *Mortierella*, a dominant fungal genus in root rot disease of Torreya grandis, significantly increases in abundance with the occurrence of plant root rot disease (Feng, 2019). In the bacterial community of healthy soil, biomarkers such as *Subgroup_7*, *MND1*, *SWB02*, and *Bradyrhizobium* were identified, with *Bradyrhizobium* capable of forming keystone ecological clusters in the rhizosphere (Fan et al., 2021) and containing functional genes related to the cycles of carbon (C), nitrogen (N), and phosphorus (P) (Hakim et al., 2021). This bacterium is effective in transforming readily degradable compounds, especially root exudates. Additionally, *Bradyrhizobium* is a plant growth-promoting rhizobium that can fix nitrogen after establishing an endosymbiotic relationship within plant nodules, and research has shown that it plays a significant role in the growth and yield increase of plants such as soybeans, peanuts, and rice (Ormeño-Orrillo and Martínez-Romero, 2019). *Subgroup_7* and *MND1* are nitrifying bacteria that play an important role in the soil nitrogen cycle by promoting nitrogen transformation and enhancing nitrogen uptake by plants, thereby improving soil fertility. Studies have shown that *MND1* is more abundant in the rhizosphere of healthy plants compared to diseased plants (Wen et al., 2020; Navarrete et al., 2015). *SWB02* has been associated with high nutrient content in soil (Cao and Fan, 2023). The enrichment of these bacterial groups may help maintain the balance and stability of the microbial community in healthy soils and enhance plant disease resistance by inhibiting the invasion of pathogenic microorganisms. Therefore, the presence of these bacterial groups may be one of the key factors enabling healthy soils to maintain higher resistance when facing diseases. In the fungal community of healthy soil, genera such as *Preussia* and *Humicol*a, which are antagonistic to pathogenic fungi, dominate. These fungi are reported to have antagonistic effects against root rot pathogens (Mapperson et al., 2014; Tao et al., 2023), particularly the genus *Humicola*. Research has demonstrated that *Humicola* can participate in suppressing pathogenic Fusarium, indicating its potential function in resisting pathogen invasion (Tao et al., 2023).

Through Mantel Test analysis, we found that the overall relationship between microbial communities (Supplementary Figure S2) in healthy and diseased soils and soil properties is consistent with the relationship between characteristic microbes (Figure 4C) and soil properties, indicating that the differences between microbial communities in healthy and diseased soils are mainly influenced by characteristic microbes. For diseased soil, there is a significant correlation between fungal characteristic microbes, AP, and *F. graminearum*, while in healthy soil, bacterial characteristic microbes are related to pH and XYL activity, and fungi are related to AP and NAG activity. Indeed, soil phosphorus content and soil pH are key determinants for microbial taxonomic groups (Banerjee et al., 2019), with phosphorus and pH showing a negative correlation with pathogen abundance and a positive correlation with beneficial microbes, especially as a higher pH value can enhance the suppression of *Fusarium* (Chen et al., 2018; Zhou et al., 2019). In healthy soil, XYL activity is primarily influenced by bacterial characteristic microbes, which mainly include Subgroup_7, MND1, *Bradyrhizobium*, etc. Meanwhile, NAG activity is dominated by fungal biomarkers such as the genus *Preussia* and *Humicola*, suggesting that these fungi are the main sources of NAG enzyme in healthy soil, contributing to the inhibition of pathogen invasion and disease development.

Multiple studies indicate a positive correlation between microbial diversity and soil multifunctionality, suggesting that higher diversity ensures better function maintenance across different environmental conditions (Wagg et al., 2014; Powell and Rillig, 2018; Delgado-Baquerizo et al., 2020; Jiao et al., 2022). This study found a significant positive correlation between soil bacterial diversity, network complexity, and characteristic microorganisms, but a significant negative correlation with the abundance of the pathogen *F. graminearum* and characteristic fungal microorganisms, with no relation to fungal diversity, which is consistent with findings by Jia et al. (2023). These results demonstrate that disease significantly disrupts the interactions between soil microorganisms and EMF. The progression of disease leads to the loss of beneficial bacteria and a rise in harmful fungi, worsening this disruption. These microbial groups significantly influence soil functions (D’Acunto et al., 2018; Nazaries et al., 2021).

## Conclusion

Our research indicates that *C. cathayensis’*s rhizosphere soil bacterial community is more sensitive to root rot compared to the fungal community, demonstrating a significant reduction in the complexity and stability of the microbial network and a stronger correlation of soil function with the bacterial community. Additionally, we observed that increased soil bacterial abundance positively influences soil versatility, whereas dominant fungal markers have a negative impact. Dominant bacteria such as *Subgroup_7*, *MND1*, *SWB02*, and *Bradyrhizobium*, along with fungal markers like *Preussia* and *Humicol* in the *C. cathayensis* rhizosphere, promote plant growth and disease inhibition, serving as potential microbial biocontrol agents against root rot. Additional fungi, including *Saitozyma*, *Arcopilus, Viridispora, Mortierella, Oidiodendron, Nigrospora*, and *Clitopilus*, were significantly enriched in the rhizosphere soil of diseased *C. cathayensis* plants. These fungi are likely associated with root rot disease and could potentially serve as microbiological indicators for assessing the health status of *C. cathayensis* rhizosphere soil. Notably, the genus *Mortierella* could serve as a potential microbial biocontrol agents against root rot disease in *C. cathayensis*.

## Data Availability

Publicly available datasets were analyzed in this study. This data can be found here: The dataset supporting the conclusions of this article is available in the NCBI Sequence Read Archive under Bioprojects (PRJNA1110578).

## Footnotes

^1^ https://github.com/taowenmicro/EasyStat

^2^ https://bio-protocol.org/bio101/MPB

^3^ https://github.com/taowenmicro/ggClusterNet
