# Corrigendum: Deciphering differences in microbial community characteristics and main factors between healthy and root rot-infected *Carya cathayensis* rhizosphere soils

**DOI:** 10.3389/fmicb.2025.1559025

**Published:** 2025-02-18

**Authors:** Wei Fang, Yiyang Zhu, Chenfei Liang, Shuai Shao, Junhui Chen, Hua Qing, Qiufang Xu

**Affiliations:** ^1^State Key Laboratory of Subtropical Silviculture, Zhejiang A&F University, Hangzhou, China; ^2^School of Environmental and Resource Sciences, Zhejiang A&F University, Hangzhou, China

**Keywords:** soil health, chemical properties, soil multifunctionality, pathogenic fungi, keystone species

In the published article, there was an error in Supplementary Figure 2 as published.

The correct version of this figure was used throughout the review process and was originally submitted during the initial manuscript submission. However, during the final submission stage, the author mistakenly uploaded the wrong version of the figure. Please note that this error only concerns Supplementary Figure 2 and the manuscript text itself remains unchanged.

The corrected Supplementary Figure 2 and its caption published online.

The authors apologize for this error and state that this does not change the scientific conclusions of the article in any way. The original article has been updated.

