# Supplementary figures and images for: Deciphering differences in microbial community characteristics and main factors between healthy and root rot-infected Carya cathayensis rhizosphere soils
Source: Front Microbiol. 2024 Nov 11;15:1448675. doi: 10.3389/fmicb.2024.1448675 (PMC11586369; doi:10.3389/fmicb.2024.1448675)

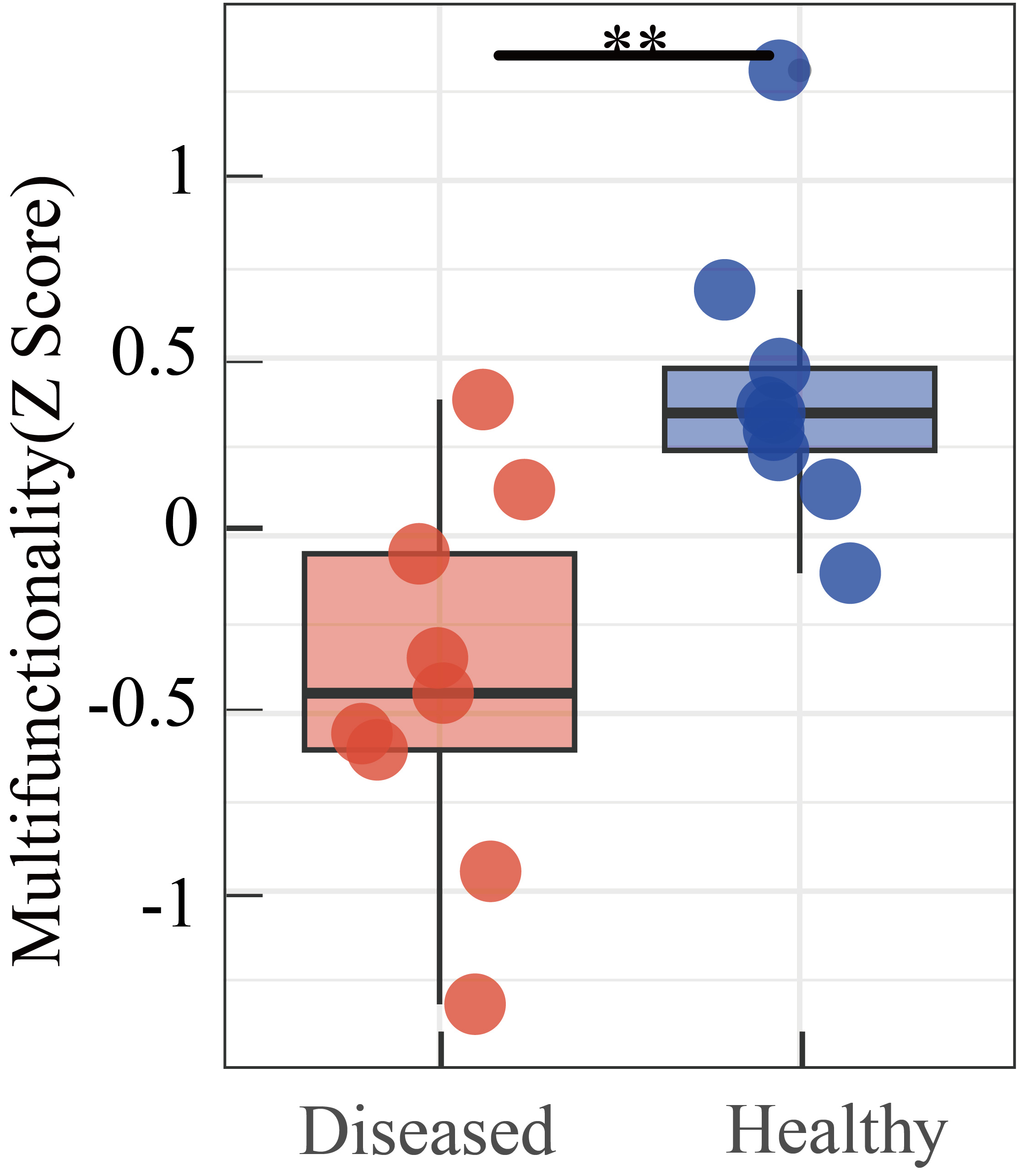

Supplement: Supplementary file 2 [file Image_1.jpg]

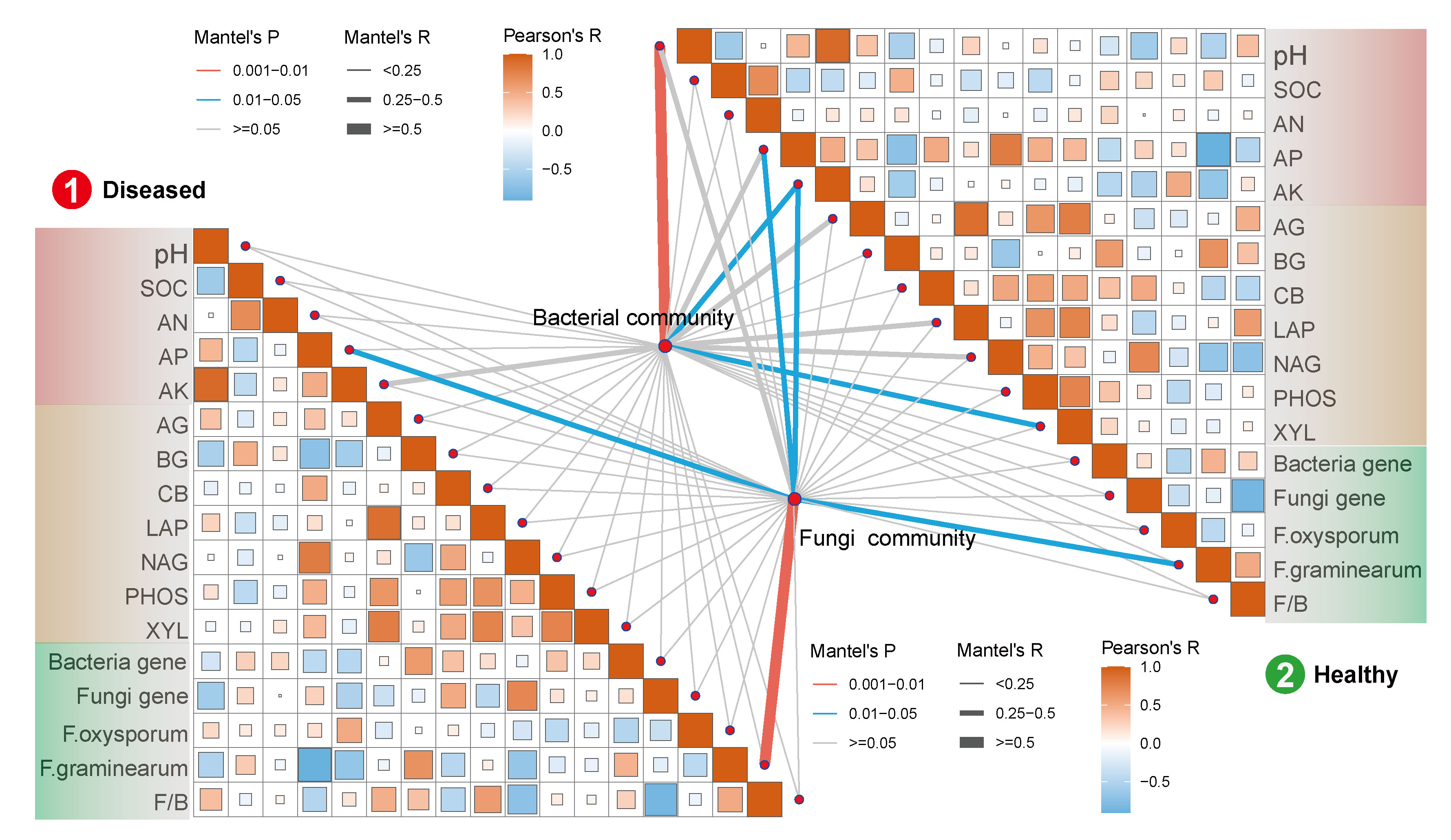

Supplement: Supplementary file 3 [file Image_2.jpeg]
